# Fractal Characterization of Non-Uniform Corrosion of Steel Bars in Concrete Beams after Accelerated depassivation and Seven-Year Natural Corrosion

**DOI:** 10.3390/ma12233919

**Published:** 2019-11-27

**Authors:** Haoyu Jiang, Nanguo Jin, Hailong Ye, Ye Tian, Xianyu Jin, Qiang Zeng, Dongming Yan, Xin Xu

**Affiliations:** 1 Department of Civil Engineering and Architecture, Zhejiang University, Hangzhou 310058, China; haoyujiang@zju.edu.cn (H.J.); jinng@zju.edu.cn (N.J.); xianyu@zju.edu.cn (X.J.); cengq14@zju.edu.cn (Q.Z.); dmyan@zju.edu.cn (D.Y.); 21912155@zju.edu.cn (X.X.); 2 Department of Civil Engineering, The University of Hong Kong, Pokfulam, Hong Kong, China; hlye@hku.hk

**Keywords:** non-uniform corrosion, seven-year outdoor corrosion, 3D laser scanning technology, fractal dimension, corrosion level

## Abstract

In this work, the non-uniform corrosion characteristics of steel bars in stressed reinforced concrete beams after accelerated depassivation and seven-year outdoor natural corrosion is analyzed using fractal theory. 3D laser scanning and 3D reconstruction technology are applied to collect the cross-sectional area along the steel bars and obtain the corrosion curves. The non-uniformity of corrosion is analyzed by fractal dimensions which is calculated by variation method. The results indicate that the initial loading level and loading zone have some influence on non-uniform characteristics of steel bars. For an ordinary concrete beam, the increase of load can cause a reduction of fractal dimension of corrosion curves by 5%, which indicates the non-uniformity of corrosion will increase with the increase of load level. The fractal dimension in the bending zone is lower than that in the tension–shear zone, which indicates that corrosion is more non-uniform in bending zone. However, the loading level and loading zone have a slight influence on corrosion level, and the maximum difference of corrosion level caused by load is merely 0.23%. Furthermore, the corrosion level increases with the decrease of fractal dimension, suggesting that the non-uniformity of corrosion increases with the growth of corrosion level. The incorporation of slag powder can help reduce the non-uniformity of corrosion, but the influence on reduction of the corrosion level is about 0.25%. For concrete structures under marine environment, application of slag powder is a good method to slow down the corrosion and reduce the non-uniformity of corrosion.

## 1. Introduction

As corrosion of steel reinforcement causes a remarkable performance deterioration of concrete structures, reliable monitoring and assessment methods of corroded reinforcement have drawn significant attention. For a long time, the steel reinforcement in concrete is assumed to be uniformly corroded, partly for simplicity of analysis [1,2]. However, it has been gradually accepted that non-uniform corrosion is the most common case of naturally corroded steel reinforcement in concrete structures in chloride-bearing environments [3,4]. The non-uniformity of corrosion along the steel reinforcement is more detrimental to the performance of concrete structures than the case of uniform corrosion [5,6]. As a result, the mechanism of non-uniform corrosion along steel reinforcement and how to reduce this non-uniformity has become a hot topic in recent years. Several mechanisms for the causes of non-uniform corrosion of steel reinforcement in concrete have been proposed, including the non-uniform spatial distribution of chloride at rebar surfaces [7,8], the stochastic nature of corrosion process [9,10], as well as the presence of cracks and defects at steel–concrete interfaces [11,12,13,14].

In order to characterize the non-uniformity of corrosion along the steel bars, lots of efforts have been made. For instances, Wang et al. defined the non-uniform corrosion coefficient R by calculating the ratio of the maximum corrosion rate to the corresponding average corrosion ratio of a corroded steel bar [15]. Considering distribution probability of pit depth and spatial variability, a similar non-uniform corrosion coefficient Rp was proposed by Stewart et al., which is defined as the maximum rust pit depth divided by the average rust pit depth [16]. In fact, the failure of a corroded steel bar usually occurs at the location with the minimum cross-sectional area. To feature this phenomenon, Zhou et al. [17] and Zhang et al. [10] used the ratio of the average cross-sectional area to the minimum cross-sectional area to describe the non-uniform corrosion coefficient R. While all these coefficients focused on the most severely corroded cross-section and the average corrosion level, such coefficients are inappropriate to distinguish the case of single pit and several pits. What is more, the fluctuation of the corroded cross-sectional area cannot be reflected. To evaluate the corrosion characteristics of a steel bar, it is necessary to find an effective method to quantify the uniformity of corrosion faithfully.

Fractal theory was developed in the 1970s, which shows its advantages in the study of complex shapes and patterns from a multi-scale perspective [18], and then has been an effective method to investigate materials properties. Many scholars have successfully applied fractal dimension to describe electrochemical noise in corrosion process, surface roughness, sphericity of sand, and damage of concrete structures [19,20,21,22,23]. In addition, an improved method was proposed by Xu et al. [24] to describe the surface profile of corroded steel bars by combining simple fractal dimension with surface roughness. The fractal theory provides a new approach to describe the self-similarity and complexity of objects and fractal dimension can be used to quantify the uniformity of corrosion with more details. The fluctuation of corroded cross-sections and the self-similarity of non-uniform corrosion can be better expressed by fractal theory. With the help of fractal theory, the factors which affect the non-uniformity of corrosion can be studied.

In this study, the fractal theory was utilized to characterize the non-uniformity of corrosion along the steel bars obtained from long-term outdoor naturally-corroded reinforced concrete beams after being dispassivated by wetting–drying cycles under different load levels. Two types of concrete, including slag concrete and ordinary Portland cement concrete, were studied to evaluate the influence of slag incorporation on the corrosion of steel bars. All the concrete beams were placed under ambient conditions for seven years to achieve the natural corrosion of steel bars. The 3D laser scanning technique was applied to obtain the morphology of cross-sections along the steel bars. The corrosion level was also calculated to evaluate the corrosion degree of steel bars. The influence of loading level, stress state, and slag incorporation on the non-uniform corrosion of steel was analyzed through fractal dimension and corrosion level analysis. In the end, the relationship between non-uniformity of corrosion and corrosion level is studied by fractal dimension for this case.

## 2. Experiment Program

### 2.1. Materials

The P.I 52.5 ordinary Portland cement (OPC) was used in this study. The fineness of OPC is 350 m^2^/kg and the corresponding mineral composition is given in Table 1. The coarse aggregate was crushed gravel with a continuous grade of 5–20 mm and the raw material was limestone. The fine aggregate was natural river sand with a fineness modulus of 2.64. The hot-rolled ribbed steel bars (HRB) 335 were utilized as longitudinal main tensile reinforcement and the hot-rolled plain steel bars (HPB) 235 were utilized as hanger steel bars and stirrups. The corresponding chemical composition of steel bars used in this experiment is given in Table 2. The yield strength and tensile strength of HRB335 are 335 and 455 MPa, respectively. While the yield strength and tensile strength of HPB235 are 235 and 370 MPa, respectively. The specific surface area of the slag powder with the fine grinding of S95 is 450 m^2^/kg, according to the Chinese standard [25]. Two types of concrete were prepared. One was made of plain OPC, which was denoted as CO. The other one was named as CS, in which 50% OPC was replaced by slag. The details of the mix proportion and the standard cube compressive strength of concrete at 28 days are shown in Table 3.

### 2.2. Specimen Preparation and Experiment Procedure

**Figure 1:**
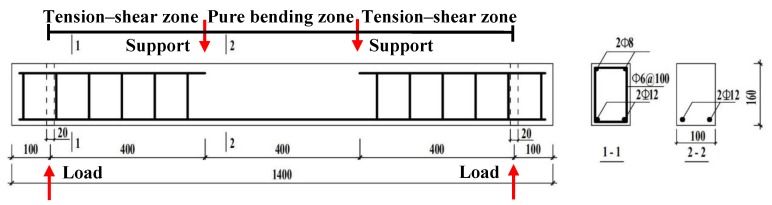
Layout of reinforcement bars [26].

**Figure 2:**
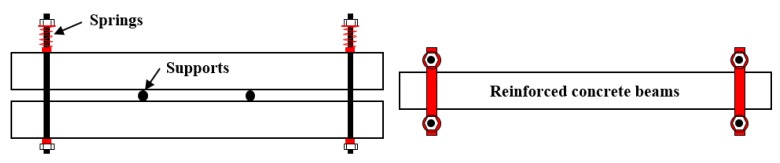
Four-point spring bending system [26].

In this work, three CO and three CS reinforced concrete beams were prepared. The size of each concrete beam was 100 × 160 × 1400 mm^3^, and the thickness of the concrete cover was 15 mm. Before casting, the steel bars were placed into a wood formwork. The diameter of each longitudinal main tensile steel rebar was 12 mm, and the diameter of hanger steel bars was 8 mm. The stirrups with a diameter of 6 mm were placed at a space of 100 mm. As mentioned above, the strength of the longitudinal main tensile reinforcement is better than that of the hanger steel bars and stirrups. It should be noted that the longitudinal main tensile reinforcement is the later research object and its material is HRB335. The layout for all steel bars in the CO and CS concrete beams was same, as shown in Figure 1 [26]. After casting, the specimens were cured following the standard curing conditions at the temperature of 20 °C and relative humidity of 90% for 24 h. Then, the specimens were demolded, and continually cured under the standard curing conditions until 28 days. Afterwards, the specimens were immersed in deionized water for 14 days to achieve full strength growth. At 42 days, the specimens were loaded in pairs with a four-point “self-stressing” spring loading system, as shown in Figure 2 [26]. The ultimate bending load is 48 kN, which is determined by bending test of beams cast from the same batch. To eliminate the influence of transverse cracks on the corrosion of longitudinal steel bars, the load levels were 0%, 10%, and 30% of the ultimate bending load to avoid macroscopic cracking, thus the non-uniformity of corrosion is mainly caused by aggregates and spatial distribution of micro-cracks. All concrete beams were divided into two groups, the CO group and the CS group, according to the concrete used in each beam.

**Figure 3:**
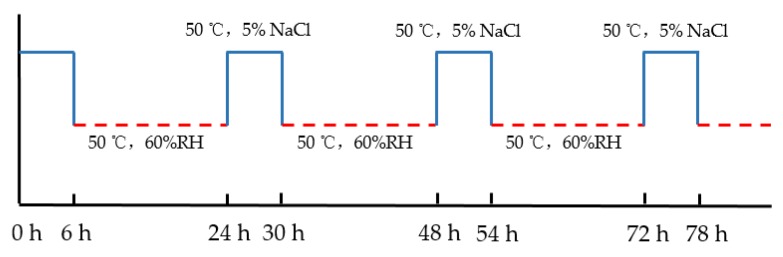
Schematic diagram of the wetting–drying cycles system.

Corrosion method plays an important role in performance degradation of steel bars and reinforced concrete members [27]. In this experiment, all steel bars were dispassivated by wetting–drying cycles at initial corrosion stage. Then all specimens were placed in an outdoor environment for natural corrosion. The specific operation process was as follows: Firstly, the concrete beams were exposed to an artificial cyclic wetting–drying condition to simulate the chloride penetration under marine environments in an environment chamber. Each wetting–drying cycle lasted for 24 h, during which the specimens were immersed in 5% NaCl solution (by weight) at 50 °C for 6 h and drying at 60% RH and 50 °C for 18 h. The schematic diagram of the wetting–drying cycles system is shown in Figure 3. The whole wetting–drying cycling process lasted for 90 days and the measured free chloride content at the depth of steel rebar ranged from 0.6% to 1.5% by weight of cement [26]. As pointed by Alonso et al., the critical chloride threshold for depassivation of steel bars was about 0.39%–1.16% [28]. As such, it was reasonable to deduce that active corrosion state of the steel bar had been induced by chloride ions in these test beams. A dial indicator with a measuring accuracy of 0.01 mm was used to continuously monitor the corrosion cracking condition of test beams. The monitoring showed that no eye-visible cracks appeared on surfaces of beams during the whole wetting–drying cycles which indicated that chloride ions transmission were only affected by internal micro-cracks, damage, and aggregate distribution. It is worth noting that the quality loss of steel bars is generally about 0.5% for a cover layer of 15 mm when corrosion expansion cracking occurs [29]. Therefore, although the corrosion level at this accelerated corrosion stage cannot be obtained with a determined value, it can be inferred that the value was smaller than 0.5%. After the accelerated corrosion, the four-point spring loading system of all concrete beams was uninstalled, and all concrete beams were naturally exposed to the outdoor environment for 7 years. The exposure location was at an urban area of Hangzhou city in Zhejiang province of China. The climate of Hangzhou belongs to a subtropical monsoon climate, where the annual average temperature is about 17 °C and the relative humidity ranges from 50% to 94% yearly.

### 2.3. Data Acquisition

After seven-year outdoor exposure, all six reinforced concrete beams were cut into two parts at the midspan. Considering the symmetry of the beam, the longitudinal steel bars were obtained from one half of each beam and the other half of the beam was reserved for further research. A total of twelve longitudinal main tensile steel bars were taken out of the beams with a length of 700 mm. To evaluate the influence of loading state on corrosion, a steel bar was cut into two parts. The part under pure bending zone and share-tension zone with a length of 600 mm was prepared for the study, which is shown in Figure 1. During the specimen preparation, the concrete on the surface of the steel bars was stripped off and the steel bars were submerged in 12% hydrochloric acid to remove the corrosion products. After pickling, the steel bars were neutralized in lime water and rinsed using water. Finally, all steel bars were placed in a dryer for four hours. In order to avoid further corrosion, steel bars were labeled and stored in anhydrous ethanol before 3D laser scanning. All the labeled steel bars are listed in Table 4.

**Figure 4:**
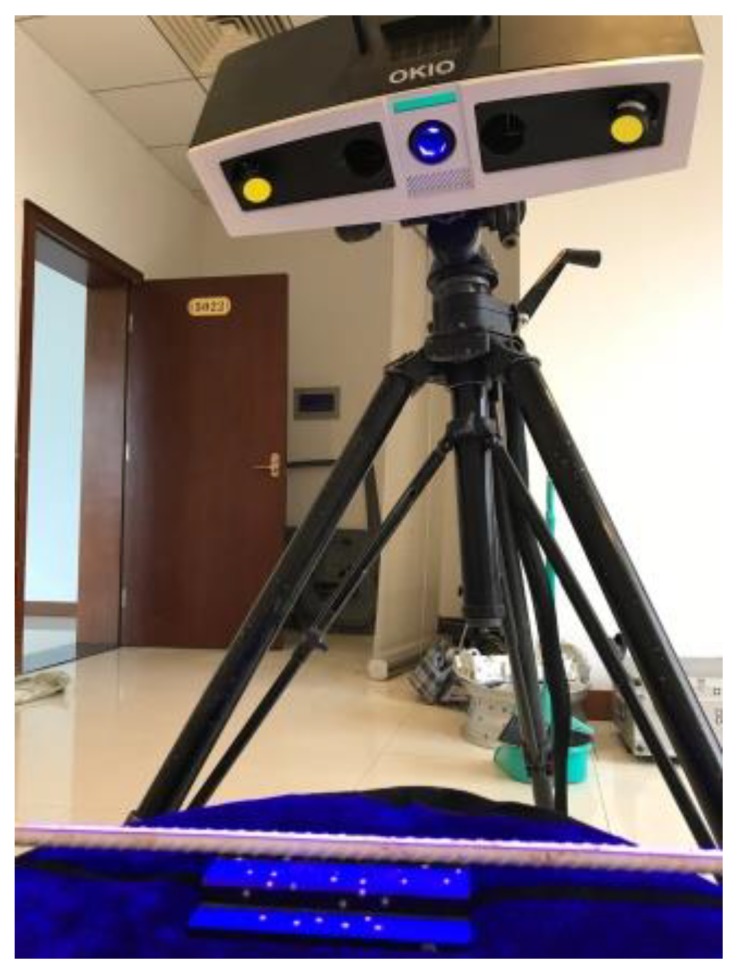
OKIO 3M laser 3D scanner.

**Figure 5:**
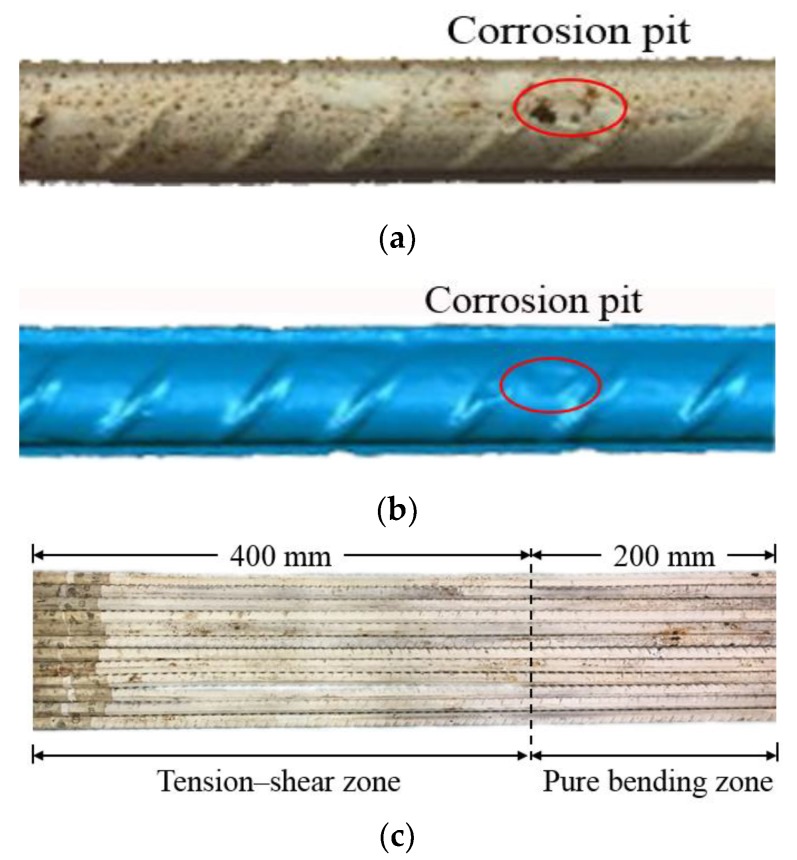
(**a**) Reconstructed solid model; (**b**) entity of the reinforcement bar; (**c**) twelve corroded steel bars.

In order to obtain the cross-sectional area data along the corroded steel bars precisely, laser 3D scanning was adopted to reconstruct their digital information. The laser 3D scanner used in this research was OKIO 3M scanner produced by Tianyuan 3d technology CO., LTD. (Beijing, China). After the optimization of point cloud data collected by the 3D scanner, a commercial software Geomagic Studio 2013 (Rock Hill, SC, USA) was adopted to reconstruct the 3D solid model. The laser 3D scanner is shown in Figure 4. A typical corroded steel bar and its restructured solid model are shown in Figure 5a,b, from which the corroded morphology can be seen in good agreement. All the twelve corroded steel bars were shown in Figure 5c, in which the steel bars can be divided into two segments: the tension–shear zone ranges from 0 to 400 mm and the pure bending zone ranges from 400 to 600 mm according to stress state.

**Figure 6:**
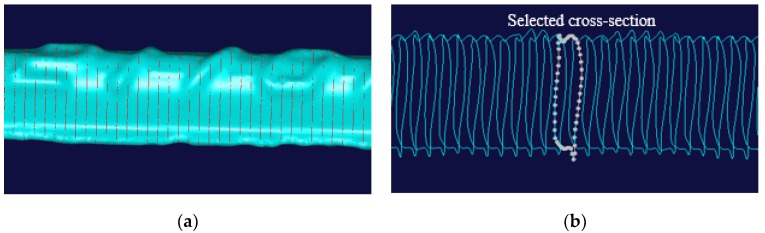
Data acquisition on the sectional area of steel bars: (**a**) Segment of the solid model; (**b**) cross-section area calculation.

The 3D models of all the steel bars were input into a commercial software NX Imageware 13 (Siemens PLM Software, Plano, TX, USA). The cross-section was recorded every millimeter along the restructured solid model of the steel bar, as shown in Figure 6a, and the corresponding area of each cross-section was calculated by UG NX 8.5, as shown in Figure 6b.

## 3. Results and Discussion

### 3.1. Cross-Section Area Distribution

**Figure 7:**
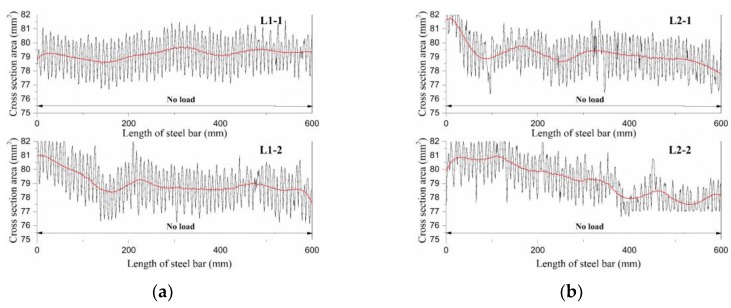

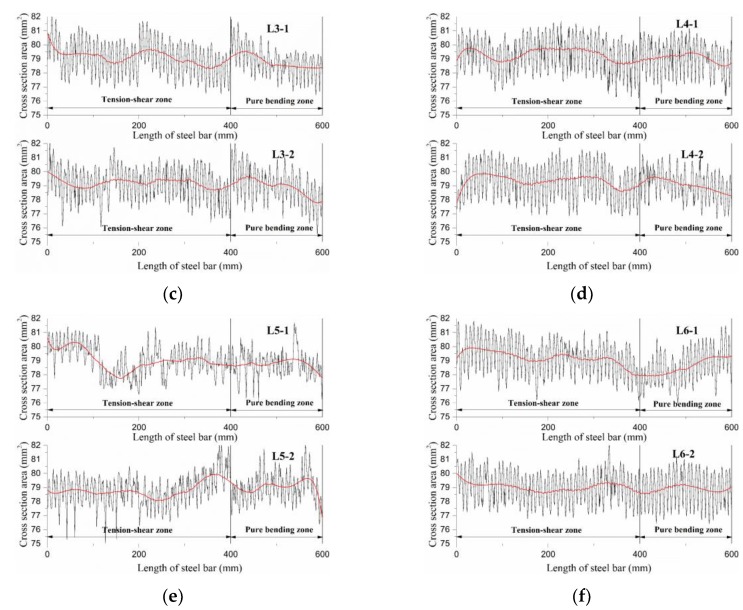
(**a**) Cross-sectional area distribution of L1; (**b**) cross-sectional area distribution of L2; (**c**) cross-sectional area distribution of L3; (**d**) cross-sectional area distribution of L4; (**e**) cross-sectional area distribution of L5; (**f**) cross-sectional area distribution of L6.

The change of cross-sectional area along the corroded steel bars are shown in Figure 7. As all the reinforcement utilized in this study are deformed steel bars, the cross-sectional area, as shown in Figure 7, fluctuates periodically along the longitudinal direction in a narrow range. The smooth fitting curves for the cross-sectional area are also plotted in red lines against the longitudinal direction of the steel bars, as shown in Figure 7. The red lines represent the average cross-sectional area of the steel reinforcements ignoring the reinforcing rib. Generally, it can be seen that the load level has influence on the corrosion level of steel bars. With the increase of load level, the smooth cross-sectional area of steel bars drops slightly, which indicates the corrosion of steel bars with a higher load level is more severe. As an evidence, in comparison with the cross-sectional area of L1 and L3, the amplitude of fluctuation for the cross-sectional area of L5 is much lower, which implies the reinforcing rib is corroded badly under a higher load level. The same conclusion can be drawn from the steel bars collected from slag concrete beams. While a comparison between the cross-sectional area of L5 and L6 demonstrates that steel bars in plain OPC concrete suffer more severe corrosion than those in slag concrete counterparts. This phenomenon supports that slag concrete has a greater resistance to chloride ion erosion [30,31,32].

**Figure 8:**
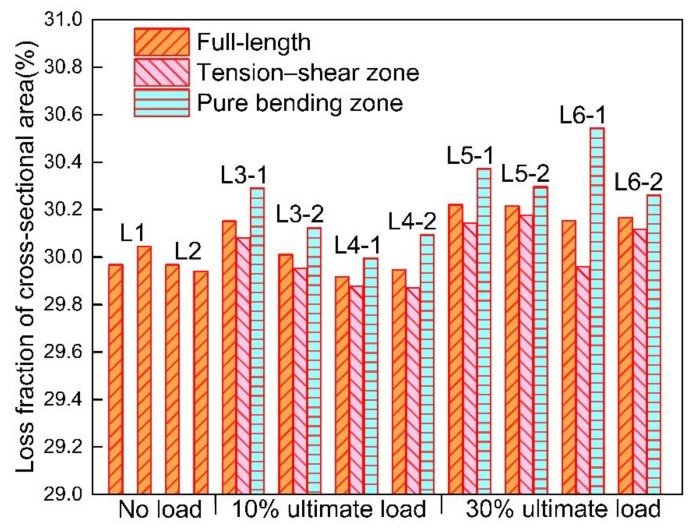
Corrosion level of each specimen.

The stress state, as shown in Figure 7, has a slight influence on the corrosion level of steel bars. The minimum and average cross-sectional area at different regions for each steel bar are given in Table 5. For L1 and L2, the minimum cross-sectional area appears randomly along the longitudinal direction of stress-free steel bars. However, the corrosion level seems more severe in regions from 400 to 600 mm than that from 0 to 400 mm in L1-2, L2-1, and L2-2, which may be caused by the randomness of corrosion. Meanwhile, the cross-sectional area of steel bars collected form L3 to L6 drops generally within the pure bending zone. The most severe corrosion is more likely to generate in the pure bending zone rather than in the tension–shear zone, as shown in Table 5. A quantitative analysis of the corroded steel bars is furtherly represented in Figure 8. And the corrosion level *η* is defined based on the loss fraction of the cross-sectional area within a length of a steel bar as Equation (1):

(1) $$\eta =1-\frac{A}{A_{0}},$$

where *A* is the average cross-sectional area within a certain length of the steel bar; *A*_0_ is the average cross-sectional area of the non-corroded steel bar.

It can be seen from Figure 8 that the corrosion level of the full-length steel bar approximately has no change when the load increases from 0% to 10% of ultimate load; however, 30% ultimate load has a slight increase with 0.23% on corrosion level. Under the same load level, the corrosion level of steel bars in group CO is mainly larger than that in group CS. It indicates that for beams incorporated with slag, the corrosion of reinforcement is inhibited. It is also proved, from Figure 8, that the corrosion levels of steel bars in the tension–shear zone are all lower than those in the pure bending zone. This phenomenon can be attributed to the fact that the concrete in the pure bending zone is in pure tension, and the concrete in the tension–shear zone subjects both tension and shear stress. The chloride ions penetrate more rapidly into concrete suffered from pure tension stress rather than that under composite stress [33]. As such, the corrosion of steel bars in the pure bending zone is more severe than in the tension–shear zone.

### 3.2. Fractal Analysis

#### 3.2.1. Fractal Dimension Calculation

Fractal dimension *D* reflects the complexity and self-similarity of the whole fractal structure, which is introduced in this study to characterize the non-uniform corrosion of a steel bar. There are a number of methods to calculate the fractal dimension, including the box counting method and variation method. The box counting method is widely accepted due to its simple arithmetic [34,35]. However, part of characteristic information of the fractal structure will be lost during the calculation, and the lost information will increase with the increase of complexity of the fractal structure [36]. In comparison to the box counting method, the variation method has the advantage of calculating the fractal dimension more accurately as it takes more detailed information of the fractal structure into consideration. Moreover, the variation method is quite suitable for fractal calculation of curve, which leads to a more accurate result. Since its first application in 1989 [37], the variation method has also been gradually utilized in many researches. The variation method is suitable for analyzing continuous function [38] and mining the local information from a complex database. What is more, the fractal dimension *D* is appropriate to analyze the complexity of the continuous signal [39], as it has been used to study the time series of pipeline with potential change [40]. Thus, the variation method was adopted to calculate the fractal dimensions of non-uniform corrosion curves in this paper. For these curves, a higher *D* indicates a higher self-similarity and more uniform corrosion.

**Figure 9:**
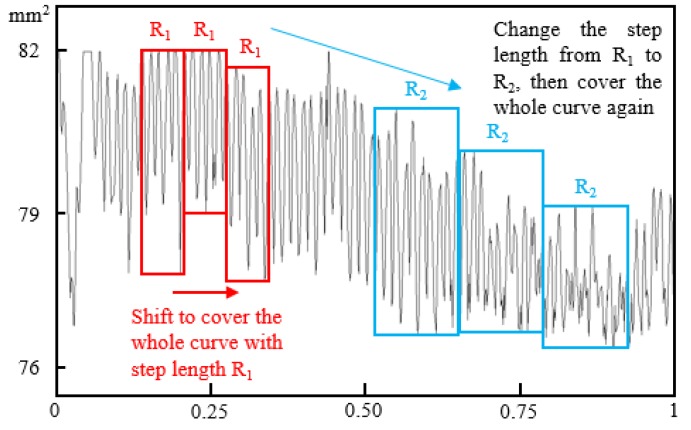
The schematic diagram of the variation method.

The algorithm of variation method is to use a rectangular window with the width of *R* to cover the given curve, the value of window width and the increment step should be carefully taken [41]. The schematic diagram is shown in Figure 9. It should be noticed that the length of the whole curve should be normalized as 1. The window moves from one end of the curve to the other end with a step length *R*, which is exactly the same as the width of the window. The height of each rectangle is determined by the amplitude difference between the highest and the lowest point of the curve in the corresponding window. Shifting the rectangular window step by step to cover the whole curve and calculating the area of each rectangular window, then the sum of the area of all shifting windows can be stated as *S*(*R*).

**Figure 10:**
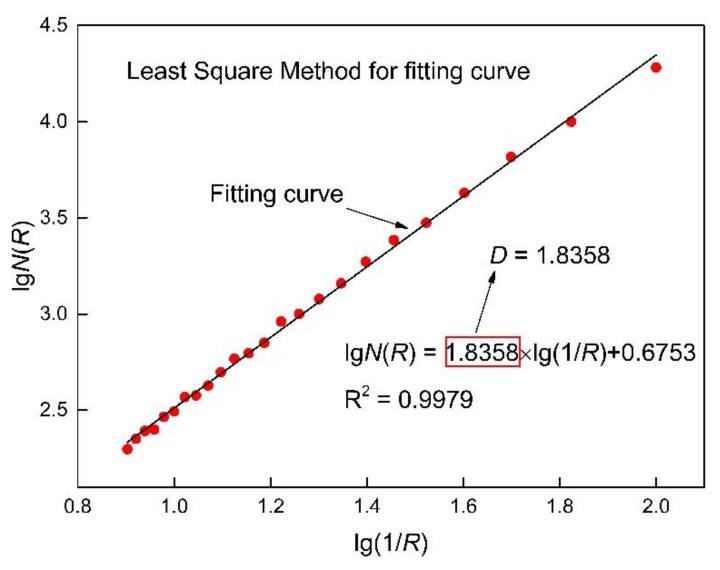
Least square method for *D* calculation.

A parameter *N*(*R*) is defined by *S*(*R*) and *R* as Equation (2). It is obvious that *N*(*R*) is a function determined by *R*. As shown in Figure 9, with a change of *R* from *R*_1_ to *R*_2_, the calculated *N*(*R*) will change from *N*(*R*_1_) to *N*(*R*_2_) correspondingly. The fractal dimension *D* can be obtained as the slope of lg*N*(*R*)~lg(1/*R*) through the least square method. As shown in Figure 10, a good linear relationship between lg*N*(*R*) and lg(1/*R*) can be recognized, which means the cross-sectional area of the corroded steel bars shows remarkable fractal features.

(2) $$N(R)=\frac{S(R)}{R^{2}},$$

where *R* is the width of the covering window, *R* ∈ [1/100, 1/2]; *S* is the sum of the area of the shifting windows.

**Figure 11:**
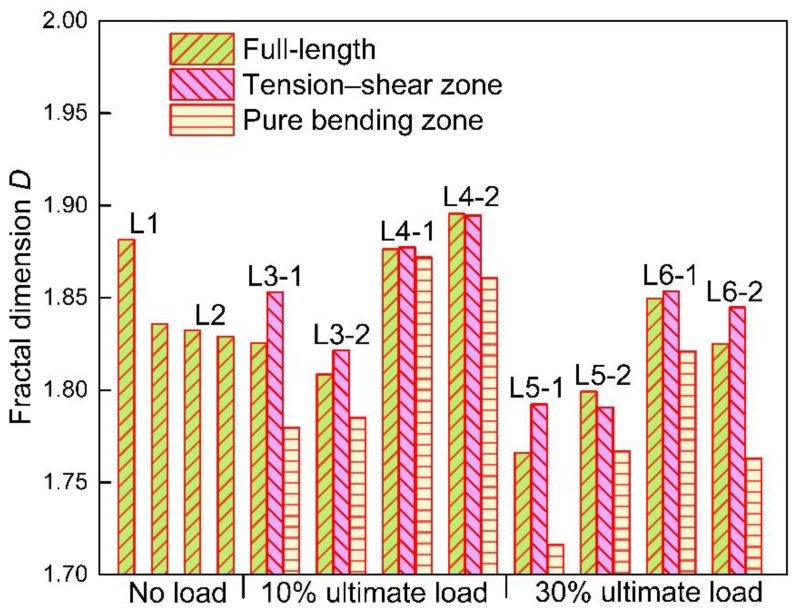
Fractal dimensions of steel bars.

According to the arithmetic of fractal dimension *D* based on the variation method, all the fractal dimensions of the cross-sectional area curves for the twelve corroded steel bars are calculated and shown in Figure 11. Generally, the fractal dimensions of steel bars obtained from the same beam are close to each other. While the fractal dimensions calculated in different concrete beams demonstrate a clear difference. This phenomenon proves that the mineral admixture and loading conditions can both influence the non-uniform corrosion of steel bars in concrete beams.

#### 3.2.2. Influence of Loading Condition on Corrosion

It can be seen from Figure 11 that the loading condition plays an important role on the non-uniform corrosion along the reinforcement bars in view of fractal dimensions. As indicated in Figure 11, the influence of loading condition on the non-uniform corrosion is reflected in two aspects (i.e., load level and stress state).

**Figure 12:**
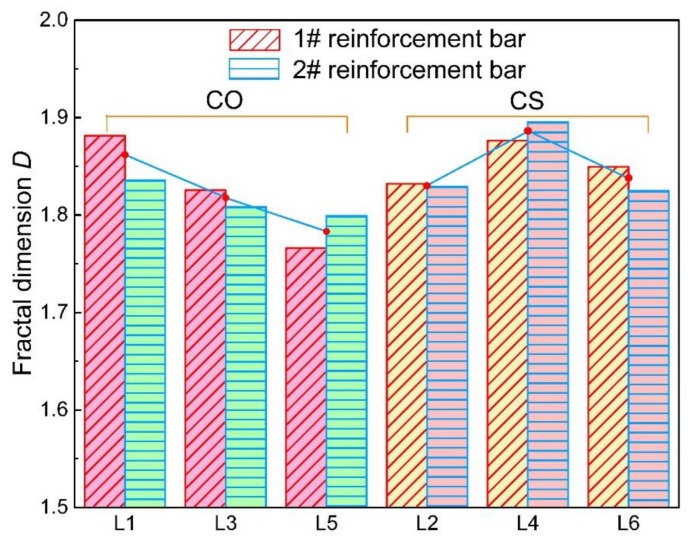
Influence of load on fractal dimension of steel bars.

The relationship between fractal dimension and load level is shown in Figure 12. As mentioned before, a higher fractal dimension represents higher self-similarity and uniformity of the corroded steel bars. It is easy to find out that the fractal dimension of group CO decreases with the increase of load level, which means the corrosion of steel bars subjected to higher load level tends to be more non-uniform along the longitudinal direction. However, it seems that the relationship between load level and fractal dimension for group CS is not so clear. The fractal dimension of full-length steel bars grows with the growth of load level from 0% to 10%, whereas it drops down from 10% to 30%. Under the initial load level of 30%, the fractal dimension changes irregularly. This phenomenon indicates that the influence of mineral admixture on corrosion of steel bars would exceed the influence of load level.

**Figure 13:**
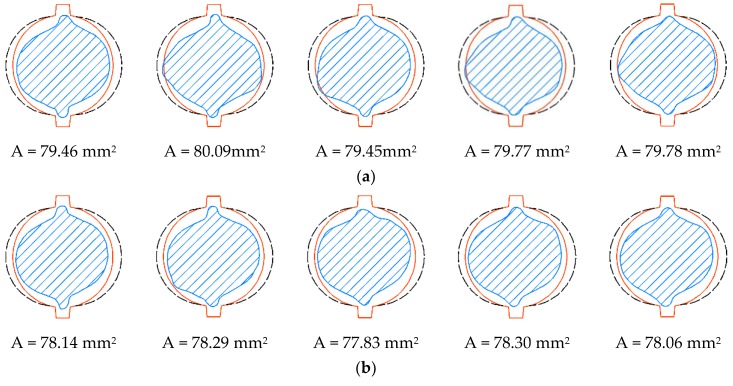
Cross-section corrosion in the pure bending zone and the tension–shear zone: (**a**) Tension–shear zone; (**b**) pure bending zone.

The concrete in the pure bending zone suffers tension stress, resulting in the micro-cracks generating vertically to the steel bars in the concrete beam. Therefore, the chloride ions will transfer to the steel bars from the surface of the beam more quickly through the shortest path. As a result, a higher free chloride ions concentration leads to more severe corrosion of the cross-section. Several typical cross-sections in these two load zones are shown in Figure 13; corresponding parts are represented by line segments of different colors. Generally, the average cross-sectional area of steel bars in pure bending zone is smaller than that in the tension–shear zone, which proves that the corrosion of steel bars in the pure bending zone is more severe. It is obvious that the right side of the ribbed steel bar is more severely corroded than the left side because it is closer to the concrete cover.

**Figure 14:**
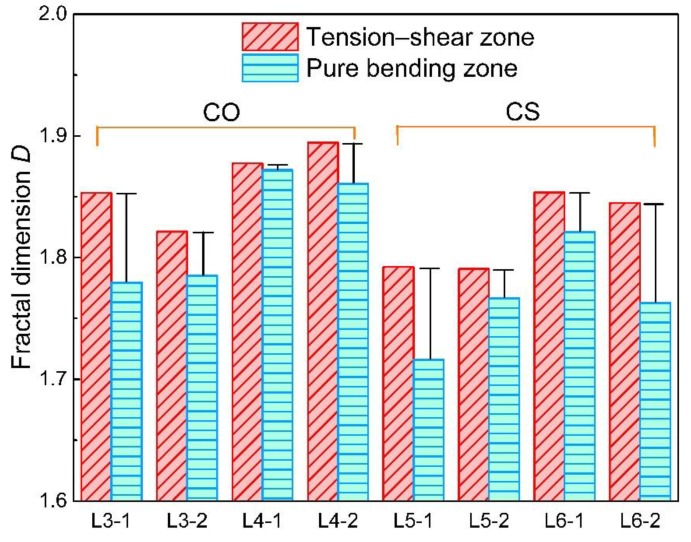
Fractal dimensions of steel bars in different stress states.

The fractal dimensions of steel bars in different loading zones are shown in Figure 11 and Figure 14. It is obvious to find out that the fractal dimensions of steel bars in the tension–shear zone are all higher than those in the pure bending zone for both CO and CS groups, which suggests that the non-uniformity of corrosion in the pure bending zone is also more remarkable.

#### 3.2.3. Influence of Slag on Non-Uniformity of Corrosion

**Figure 15:**
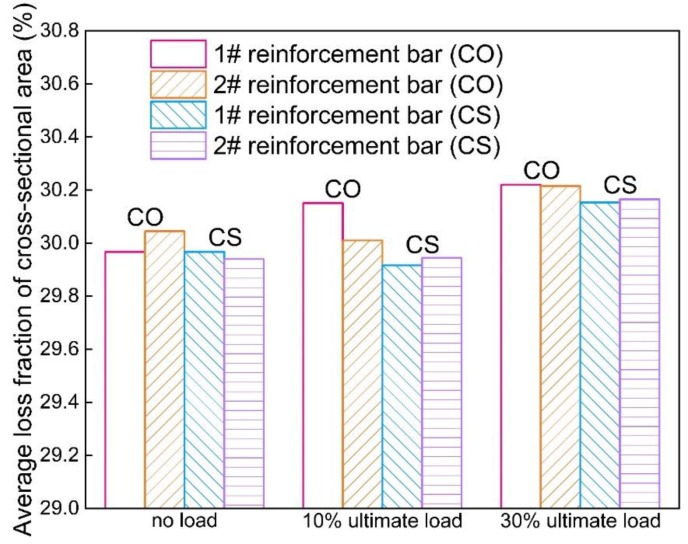
Corrosion level of full-length steel bars.

The corrosion level of the full-length steel bars under the same load level also exhibit a difference, as shown in Figure 15. It is found that the incorporation of slag helps to reduce the corrosion level of steel bars in reinforced concrete. Although the maximum decease of corrosion level caused by the incorporation of slag is merely 0.25% under the same initial load. In this case, the corrosion level is pretty high to reach approximately 30%, and the effect of slag is small. However, the slag also plays a role in weakening the corrosion level. This can be attributed to the pozzolanic reaction caused by slag that enhances the compactness of a concrete structure. That is to say, slag replacement reduces the invasion of oxygen and chloride ions. What is more, the incorporation of slag helps to recover the micro-cracks caused by initial loads, which will also reduce the non-uniform corrosion of steel bars embedded in concrete beams. It can also be seen in Figure 11 and Figure 14 that, under the same load level, the non-uniformity of the CO group is higher than that of the CS group. This phenomenon indicates that the slag incorporation restrains the non-uniformity of corrosion of steel bars. For severe environments, such as the marine environment, adding slag powder is a good way to slow down the corrosion process and reduce the non-uniformity of corrosion. The fractal dimension of L2 is abnormally low. It may be attributed to the small sample size or the fluctuation of fractal dimension under different load magnitudes caused by the randomness of corrosion.

#### 3.2.4. Relationship between Corrosion Rate and Fractal Dimension

It can be concluded from Figure 8 and Figure 11 that the non-uniformity is, to a certain extent, related with the corrosion level of steel bars. A higher corrosion level of steel bars, for both CO and CS groups and under different load levels, corresponds to a higher non-uniformity of corrosion. This conclusion is consistent with the results concluded by Zhang et al [9,10]. In this research, the concentration of chloride ions on the surface of steel bars is the main cause inducing corrosion. For chloride ions diffusion in concrete, the ITZ effect induced by coarse aggregate will improve the permeability of concrete, while the tortuosity effect will reduce permeability reversely [42]. Therefore, the random distribution of aggregates and micro-cracks cause the distribution of chloride ions in concrete accordingly and leading to the non-uniform corrosion. As such, the corrosion of steel bars shows remarkable non-uniformity with an increase of corrosion level correspondingly.

**Figure 16:**
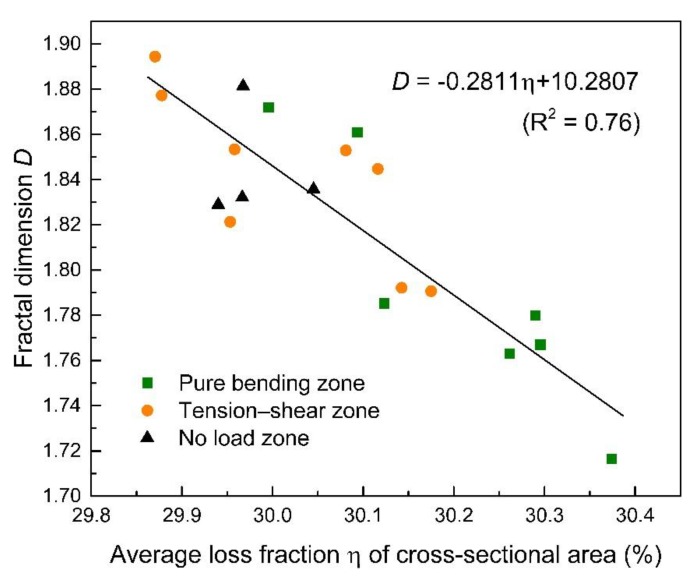
Correlation between corrosion level and corresponding fractal dimension.

A quantitative analysis on the corrosion level and the fraction dimension of steel bars is further performed, as demonstrated in Figure 16. It shows that there is a linear relationship between these two variables, which supports that the non-uniformity of corrosion increases with the increase of corrosion level [10]. In addition, it should be noticed that the fractal dimensions and corrosion levels of steel bars in the no load zone, the pure bending zone, and the tension–shear zone all follow the similar trend. This result suggests no matter what load level and stress state the steel bars suffers, the non-uniformity of corrosion is essentially correlated with the corrosion level. However, the corrosion level is pretty high, from 29.8% to 30.4%, in this case, and this relationship may not be suitable for different corrosion levels.

## 4. Conclusions

In this paper, the non-uniform corrosion characteristics of reinforced concrete beams after depassivation under different load levels and seven-year outdoor natural corrosion is studied by fractal dimension. The following conclusions can be drawn based on this study:

1. The non-uniformity of corrosion along a reinforcement bar can be expressed by the fractal dimension; the smaller the fractal dimension is, the higher the non-uniform corrosion degree will be.
2. The load level has a slight influence on corrosion level and it has some influence on non-uniform corrosion along the reinforcement bar embedded in the ordinary reinforced concrete beam. A larger load level will result in a higher corrosion level for the CO group. Meanwhile, the incorporation of mineral powder helps to reduce the corrosion level and the non-uniformity of corrosion.
3. The loading zone leads to different stress states, which induces non-uniform corrosion along the reinforcement bar. The average loss fraction of cross-sectional area and non-uniform corrosion degree in the pure bending tension zone are generally higher than that in the tension–shear zone.
4. In this case, there is a positive proportion correlation between the average loss fraction of cross-sectional area and the non-uniformity of corrosion along the reinforcement bar.

## Figures and Tables

**Table 1:** Mineral composition of cement (mass%).

Mineral Composition	C_3_S	C_2_S	C_3_A	C_4_AF	Gypsum
Content	55.5	19.1	6.5	10.1	5.0

**Table 2:** Trace element content in steel bars (mass%).

Composition	C	Si	Mn	P	S	Ceq ^1^
HRB335	0.25	0.80	1.60	0.045	0.045	0.52
HPB235	0.22	0.30	0.65	-	-	-

^1^ Carbon equivalent.

**Table 3:** Mix proportions and compressive strength of concrete (kg/m^3^).

ID	Water-to-Binder Ratio	Cement	Slag	Water	Fine Aggregate	Coarse Aggregate	Compressive Strength (MPa)
CO	0.53	370	0	188	750	1112	50.6
CS	0.53	185	185	188	750	1112	45.9

**Table 4:** Serial number of corroded reinforcement bars.

Concrete Type	CO	CS	CO	CS	CO	CS
Proportion of ultimate load Specimen label	0	0	10%	10%	30%	30%
	L1-1	L2-1	L3-1	L4-1	L5-1	L6-1
	L1-2	L2-2	L3-2	L4-2	L5-2	L6-2

**Table 5:** Minimum and average cross-sectional area.

**Specimen Label**	**L1-1**	**L1-2**	**L3-1**	**L3-2**	**L5-1**	**L5-2**
Minimum area for 0 to 400 mm	76.6446	76.4769	76.5054	76.0669	76.5907	74.9835
Average area for 0 to 400 mm	79.1380	79.8435	79.0787	79.2229	79.0090	78.9721
Minimum area for 400 to 600 mm	77.0756	76.2574	76.4212	75.5797	76.0123	76.2791
Average area for 400 to 600 mm	79.3444	77.6694	78.8420	79.0306	78.7470	78.8357
**Specimen Label**	**L2-1**	**L2-2**	**L4-1**	**L4-2**	**L6-1**	**L6-2**
Minimum area for 0 to 400 mm	76.3492	76.6179	76.5348	76.8448	76.1551	76.7400
Average area for 0 to 400 mm	79.4435	79.8534	79.3082	79.3085	79.2173	79.0386
Minimum area for 400 to 600 mm	76.3812	76.9876	76.2062	76.6481	75.9862	76.3994
Average area for 400 to 600 mm	78.7362	78.0063	79.1747	79.0640	78.5557	78.8741
